# Gestational Vitamin 25(OH)D Status as a Risk Factor for Receptive Language Development: A 24-Month, Longitudinal, Observational Study

**DOI:** 10.3390/nu7125499

**Published:** 2015-12-02

**Authors:** Frances A. Tylavsky, Mehmet Kocak, Laura E. Murphy, J. Carolyn Graff, Frederick B. Palmer, Eszter Völgyi, Alicia M. Diaz-Thomas, Robert J. Ferry

**Affiliations:** 1 Department of Preventive Medicine, University of Tennessee Health Science Center, 66 N. Pauline Street, Memphis, TN 38163-2181, USA; mkocak1@uthsc.edu (M.K.); evolgyi@uthsc.edu (E.V.); 2 Urban Child Institute, 600 Jefferson Avenue, Memphis, TN 38105, USA; 3 Department of Psychiatry, University of Tennessee Health Science Center, 711 Jefferson Avenue, Memphis, TN 38163, USA; lmurphy@uthsc.edu; 4 Boling Center for Developmental Disabilities, University of Tennessee Health Science Center, 711 Jefferson Avenue, Memphis, TN 38163-2167, USA; jgraff@uthsc.edu (J.C.G.); fpalmer@uthsc.edu (F.B.P.); 5 College of Nursing, University of Tennessee Health Science Center, 711 Jefferson Avenue, Memphis, TN 38163, USA; 6 Department of Pediatrics, University of Tennessee Health Science Center, 50 N. Dunlap Street, Memphis, TN 38103-2893, USA; 7 Division of Pediatric Endocrinology, Department of Pediatrics, University of Tennessee Health Science Center, 50 N. Dunlap Street, Memphis, TN 38103-2800, USA; adiaztho@uthsc.edu; 8 Department of Psychology, University of Memphis, 352 Psychology Building, Memphis, TN 38152-3370, USA

**Keywords:** vitamin D, language development, cognitive development, prenatal nutrition, CANDLE study, gestation

## Abstract

Emerging data suggest that vitamin D status during childhood and adolescence can affect neurocognitive development. The purpose of this study was to investigate whether gestational 25(OH)D status is associated with early childhood cognitive and receptive language development. The Conditions Affecting Neurocognitive Development and Learning in Early Childhood Study (CANDLE) study enrolled 1503 mother-child dyads during the second trimester of healthy singleton pregnancies from Shelby County TN. Among 1020 participants of the total CANDLE cohort for whom 25(OH)D levels were available, mean gestational 25(OH)D level during the second trimester was 22.3 ng/mL (range 5.9–68.4), with 41.7% of values <20 ng/dL. Cognitive and language scaled scores increased in a stair-step manner as gestational 25(OH)D levels in the second trimester rose from <20 ng/dL, through 20–29.99 ng/dL, to ≥30 ng/dL. When controlling for socioeconomic status, race, use of tobacco products, gestational age of the child at birth, and age at the 2-year assessment, the gestational 25(OH)D was positively related to receptive language development (*p* < 0.017), but not cognitive or expressive language.

## 1. Introduction

Brain development begins close to conception and continues beyond early childhood. Micro- and macronutrients have been shown to affect neurocognitive development in early childhood, with long-term consequences for academic achievement, employment, and health status [1,2]. Of many micronutrients studied, roles have been emerging for gestational 25-hydroxyvitamin D [25(OH)D] status with respect to cognitive and language development during early childhood [3,4,5].

Vitamin D is a pleiotropic secosteroid hormone, which is ingested or produced by skin exposure to ultraviolet light [6,7]. Vitamin D exerts classical actions on bone mass and non-classical effects on non-skeletal tissues throughout life [8,9]. While thresholds for vitamin D sufficiency have been debated recently [10,11], low circulating levels of 25(OH)D have been associated with increased mortality in black and white older adults dwelling in North America [12]. Recent studies have suggested significant impact by gestational 25(OH)D status upon placental function [13] and early brain development [14,15,16,17,18].

Compared to European-Americans (EA), lower 25(OH)D levels during pregnancy and altered 25(OH)D skeletal metabolism in African-Americans (AA) are well documented [10,19]. Biologically, higher melanin pigment in dermis of individuals with African ancestry contributes to lower production of 25(OH)D, yet higher bioavailable 1,25(OH)_2_-D circulates due to lower levels of vitamin D binding protein (VDBP) [10]. Unlike hepatocytes, neurons express megalin [20]. Megalin is a transmembrane protein that internalizes free 25(OH)D from its circulating complexes with VDBP and albumin. This transport mechanism, along with other renal and skeletal adaptations by AA [21,22,23], may buffer the developing brain from ambient vitamin D insufficiency. However, numerous factors have been associated with neurocognitive development and could possibly confound an association between gestational 25(OH)D and neurocognitive development, including preterm birth, socioeconomic status, and gestational exposures to maternal use of alcohol or tobacco [24,25,26].

The Conditions Affecting Neurocognitive Development and Learning in Early Childhood (CANDLE) Study is a contemporary birth cohort representing a racially and socioeconomically diverse, North American, metropolitan population [27]. The primary aim of the CANDLE study was to determine the pre- and postnatal factors that impact neurocognitive development by age 3. This report examines the hypothesis that gestational 25(OH)D status is associated with cognitive and language development at age 2 years. A secondary aim is to examine whether differences in gestational 25(OH)D and other socio-economic indicators could explain some well-recognized differences in neurocognitive scores between AA and EA [28,29].

## 2. Materials and Methods

### 2.1. Study Population

Between 2006 and 2011 the CANDLE study enrolled 1503 healthy women during their second trimester of pregnancy [27,30]. As we reported previously in *Nutrients* [30], inclusion criteria included women ages 16 to 40 years, residents of Shelby County TN, ability to speak and understand English, and 16–28 weeks of gestation with singleton pregnancy. Informed consent was given by participants 18 years or older, and assent was given by those 16–17.9 years, with consent provided by their legally authorized representative prior to enrollment.

**Figure 1:**
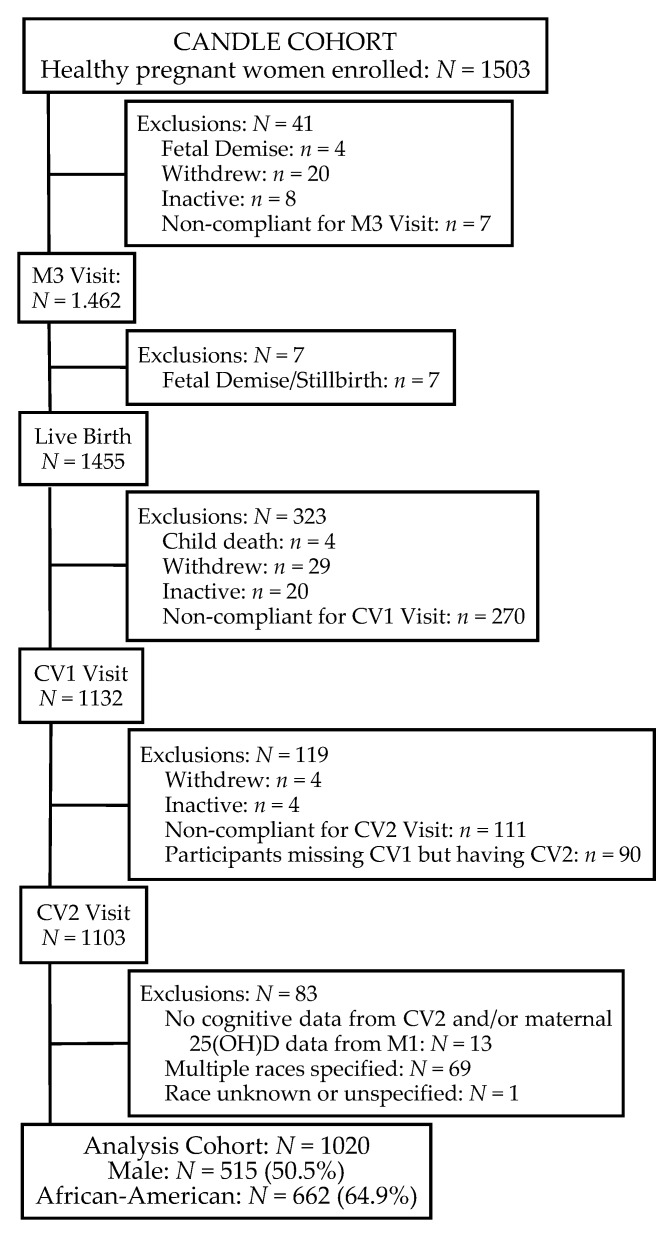
CANDLE participants available for study. M3, maternal visit 3; CV1, child visit 1; CV2, child visit 2.

Exclusion criteria included: an existing chronic disease requiring medication (e.g., hypertension, type 1 or type 2 diabetes mellitus, sickle cell disease or trait, renal disease, hepatitis, lupus erythematous, scleroderma, pulmonary disease, heart disease, human immunodeficiency virus); pregnancy complications (including maternal red cell alloimmunization though Rh factor incompatibility was permitted, prolapsed or ruptured membranes, oligohydramnios, complete placenta previa); and not intending to deliver at one of four participating hospitals [30]. The study was conducted in accordance with the Helsinki Declaration and approved by the Institutional Review Board of The University of Tennessee Health Science Center. Of the 1455 live births, 1020 mother-child dyads completed the 2-year clinic exam and had mothers who provided blood for 25(OH)D levels during the second trimester. Figure 1 outlines the participants available for this study.

### 2.2. Maternal Measures

Trained research staff orally queried baseline maternal factors and entered the data directly into the CANDLE study database. Participants self-reported ethnicity and race, highest educational level achieved (for analyses we grouped those who completed high school or less), age, health insurance type as a proxy for household income (*i.e.*, private *vs.* Medicaid/Medicare), marital status (married/single), parity (primiparous/multiparous), pre-pregnancy body mass index (BMI), cigarette smoking during pregnancy (Yes/No), alcohol use during pregnancy (Yes/No), and year of initial enrollment (2006–2011). Pre-pregnancy BMI (kg/m^2^) was calculated from self-reported height and weight prior to pregnancy, data which were collected at enrollment. Trained developmental psychologists administered the Wechsler Abbreviated Scale of Intelligence (WASI) [31] to each participating CANDLE mother at the 2-year visit (child visit CV2). During the second trimester of pregnancy (maternal visit M1 visit at 16–26 weeks’ gestation), trained research staff administered the Block Food Frequency Questionnaire (FFQ), which captured the mothers’ nutritional intake during the previous three months, including dietary supplements [30]. We excluded respondents who reported implausibly low (<1000 kcal/day) or high (>5000 kcal/day) total energy intake (*n* = 93). Willett and colleagues [32] reported using an allowable energy range of 500–3500 kcal/day for non-pregnant, non-lactating women, which we previously adapted for the increased energy needs of pregnancy [30].

### 2.3. Child Measures

The National Health and Nutrition Examination Survey guided anthropometry measurements [33]. Gestational age was determined either by ultrasound (58%), by mother’s report of the last day of menstrual cycle (37.3%), estimated by the first day of the menstrual cycle (3.4%), or by unknown methods (1.3%). Toddler weight and standing height were recorded at age 2 years, and percentiles were calculated based on the Centers for Disease Control and Prevention growth standards. Child cognitive development was assessed at the 2-year visit (CV2) using the Bayley Scales of Infant and Toddler Development^®^, 3rd Edition (Bayley-III^®^) (Harcourt Assessment, Inc., San Antonio, TX, USA) [34]. As stated in the Bayley-III manual [34], “The Cognitive Scale includes items that assess exploration and manipulation, object relatedness, concept formation, memory, and other aspects of cognitive processing.” The Receptive Communication subtest includes items that assess verbal comprehension, pronouns, being able to identify objects and pictures that are requested, and social referencing. The Expressive Communication subtest includes items that assess vocabulary development and the naming of objects, pictured objects, and pictured actions. The list of specific items administered is available on request. Use of scaled scores provided an acceptable metric for Bayley-III intra-test comparisons, while also providing a convenient measure to compare to gestational 25(OH)D.

### 2.4. Vitamin 25(OH)D Measurement

Plasma was collected casually from a free-flowing peripheral vein during the second trimester, transported on ice, then divided in aliquots prior to freezing at −20 °C within 6 h of collection. Vitamin D has proven stable in serum and plasma at room temperature before freezing for up to 72 h, and unaffected by multiple freeze/thaw cycles [35,36]. After batch thawing, plasma 25(OH)D levels were quantified by commercial enzymatic immunoassay (IDS, Boldon, Tyne and Wear, UK), performed according to the manufacturer’s instructions in a laboratory that participates in the College of American Pathology Quality Assessment Program for 25(OH)D assays. Minimum detection range of this assay was 2 ng/mL, with interassay variability <6% and precision within 1 SD of mean, using NIST SRM972 as standard.

### 2.5. Data Analysis

All analyses were performed using SAS v9.4 (SAS Institute, Inc., Cary, NC, USA). Descriptive statistics summarized demographic, maternal, and child characteristics in the data by race categories. European American (EA) excluded all races other than white, due to the small number of other races and multiple races, *i.e.*, *n* = 98 non-white, non-African-American (non-AA) in CANDLE overall.

To investigate correlations between any two continuous variables of interest, we estimated Spearman’s rank correlation. Association between two categorical variables of interest was assessed using the chi-squared test or Fisher’s exact test for 2 × 2 tables when applicable. Distribution of a continuous variable of interest was compared across different levels of a categorical variable using the Wilcoxon signed-rank test, or its multinomial extension, the Kruskal-Wallis test. *p* < 0.05 was considered statistically significant for all tests.

We recognized that discrepancies in 25(OH)D status between the AA and EA groups were likely to reveal relationships and sought to identify a cutoff for such effects without *a priori* assumptions about such cutoffs for either the AA group or the EA group. 25(OH)D was used as a continuous variable in our models, but the model estimates were obtained for 10-unit increase (based on clinical significance) rather than 1-unit increase, which are simply the 10-times inflated versions of estimates based on 1-unit increase. Estimates of Pearson and Spearman correlation coefficients were virtually the same (data not shown). However, we reported the Spearman for more robust results with respect to outliers and even slight departure from normality assumption.

Associations between select variables suspected to affect both the outcomes (*i.e.*, cognitive and language scaled scores) and the independent variable, 25(OH)D, were performed to assess possible confounders. 25(OH)D status at enrollment during the second trimester strongly correlated with 25(OH)D status at delivery (*r* = 0.84, *p* < 0.001). Since more 25(OH)D values were available during mid-gestation for analysis, we used only gestational 25(OH)D at enrollment as the main predictor of neurocognitive development.

Given known metabolic differences and distribution differences in 25(OH)D metabolism between racial groups [10], we tested for possible interaction between 25(OH)D and racial groups on scaled cognitive and language scores. Based on supplemental studies of vitamin D during pregnancy, a 10 ng/mL increase in 25(OH)D has resulted in a protective odds ratio for healthy pregnancy outcomes [37]; thus, we assessed such effects on cognitive and language scaled scores. Our modeling approach determined those variables which were significantly related to cognitive and language scaled scores. Using stepwise selection (0.20 for entry, 0.05 to stay) to remove the insignificant variables, we retained 25(OH)D status in the model while assessing the following possible confounders: race (AA or EA), maternal IQ, use of tobacco products or alcohol during pregnancy, maternal dietary intake of vitamin D, age, pre-pregnancy BMI, total number of completed pregnancies, gestational hypertension or gestational diabetes, insurance status, level of maternal education, marital status, child’s age at CV2, and gestational age at birth.

## 3. Results

### 3.1. Description of the Study Participants

The participants reflected the demographics of Shelby County, Tennessee. CANDLE mothers were more likely to report African ancestry, have a high school degree or lower, or live with a partner (Table 1). The sample was almost equally divided between Medicaid/Medicare insurance and other types of medical insurance, a proxy indicator of economic resources and income. At enrollment, 30.3% of the mothers reported the pregnancy to be their first, 11.5% reported delivery of 6 or more children, and the remaining participants reported delivery of between 2 and 5 children. Rates of smoking were 10.2% and 7.3% for EA *vs.* AA, respectively. Similarly, rates of alcohol use were 14.3% *vs.* 5.2% for EA *vs.* AA, respectively. The majority of participants displayed gestational 25(OH)D level below 30 ng/dL (90% of AA and 75% of EA, data not shown). Mean gestational 25(OH)D levels were lower for AA (20.4 ± 7.5 ng/dL) compared to EA (25.8 ± 9.0 ng/dL) (*p* < 0.0001; data not shown). Median dietary intake of vitamin D during the second trimester for the whole cohort was 177 IU (range 13.3–772).

**Table 1:** Characteristics of the Mothers at Enrollment and the Children at 2-year Assessment (CV2; *n* = 1020).

	*n*	%
Insurance		
Other Insurance	489	47.9
Medicaid, Medicare	531	52.1
Education		
HS diploma or lower	567	55.6
College or higher degree	453	44.4
Marital Status		
Cohabitation	601	58.9
Single	419	41.1
Pre-Pregnancy BMI, kg/m^2^		
Underweight	45	4.4
Normal	397	38.9
Overweight	240	23.5
Obese	338	33.1
Alcohol Use, Yes	86	8.4
Tobacco Use, Yes	85	8.3
Total Pregnancies ^1^		
1	309	30.3
2–5	594	58.2
>5	117	11.5
Gestational 25(OH)D		
<20 ng/dL	425	41.7
20.00–29.99 ng/dL	440	43.1
≥30 ng/dL	155	15.2
	**Mean**	**SD**
Weeks of Gestation	38.9	1.7
25(OH)D, ng/dL	22.3	8.5
Maternal Age, years	26.6	5.5
Maternal Total IQ	96.2	16.4
Birth Weight, *Z*-score	0.05	0.95
Birth Length, *Z*-score	0.53	1.28
Head Circumference, *Z*-score	−0.03	1.34
Child Age at CV2 Assessment, months	25.0	1.5
Cognitive Scaled Score ^2^	9.6	2.6
Receptive Language Scaled Score ^2^	9.5	2.9
Expressive Language Scaled Score ^2^	9.9	2.7

^1^ Includes current pregnancy and previously completed term and pre-term pregnancies; ^2^ As assessed at 2-year exam (CV2).

At the 2-year assessment (CV2), the average age of the children was 25 months, and their mothers were in their mid-twenties. Fifty-one percent of these children were male, with mean gestational age at birth of 38.9 weeks. 8.0% of children were born pre-term before 38 weeks gestation. Birth weights/lengths of the CANDLE children were recently reported in *Nutrients* [38].

### 3.2. Associations between Select Characteristics and Cognitive and Language Scaled Scores

Table 2 presents the children’s scaled developmental scores by selected factors. Generally, factors that indicated lower socioeconomic status resulted in lower standard scaled scores for those on Medicaid/Medicare, those with a high school degree or lower, and mothers who reported to be single. Compared to non-users, individuals who used tobacco products during pregnancy had offspring with lower scaled scores. As the number of pregnancies increased, the scaled scores declined. As expected, pre-term children displayed lower scaled scores. Gestational diabetes had no observed impact on scaled scores, while those who developed pre-eclampsia had lower scaled scores. As gestational 25(OH)D level increased, so did the scaled scores. Dietary intake of vitamin D was not associated with scaled scores or mid-gestational 25(OH)D levels.

**Table 2:** Scaled Developmental Scores by Selective Characteristics (*n* = 1020).

	Cognitive			Receptive Language			Expressive Language		
	Mean	SD	*p* ^1^	Mean	SD	*p* ^1^	Mean	SD	*p* ^1^
Race			<0.0001			<0.0001			<0.0001
African-American	8.8	2.2		8.6	2.3		9.3	2.3	
European-American	11.0	2.7		11.1	3.1		11.0	3.1	
Insurance			<0.0001			<0.0001			<0.0001
Other Insurance	10.4	2.8		10.5	3.1		10.7	3.0	
Medicaid, Medicare	8.8	2.1		8.6	2.3		9.2	2.2	
Education			<0.0001			<0.0001			<0.0001
HS diploma or lower	8.9	2.1		8.7	2.4		9.3	2.2	
College or higher degree	10.4	2.8		10.5	3.1		10.7	3.0	
Marital Status			<0.0001			<0.0001			<0.0001
Cohabitation	10.1	2.7		10.1	3.0		10.3	2.9	
Single	8.8	2.2		8.6	2.3		9.3	2.3	
Pre-Pregnancy BMI, kg/m^2^			0.0001			0.0001			0.0001
Under weight	9.7	2.2		9.9	2.9		9.9	2.6	
Normal	9.9	2.7		9.9	3.1		10.4	2.9	
Overweight	9.5	2.3		9.4	2.7		10.0	2.6	
Obese	9.2	2.6		9.0	2.7		9.4	2.4	
Alcohol Use			0.0024			0.002			0.007
No	9.5	2.5		9.4	2.8		9.8	2.6	
Yes	10.3	3.0		10.7	3.6		10.6	3.1	
Tobacco Use			<0.0006			<0.0006			<0.0006
No	9.6	2.6		9.6	2.9		10.0	2.7	
Yes	8.7	2.0		8.5	2.7		8.8	2.6	
Total Pregnancies			0.0006			0.029			0.0028
1	10.0	2.9		9.8	3.0		10.3	2.7	
2–5	9.5	2.4		9.5	2.9		9.8	2.8	
>5	8.8	2.2		8.8	2.3		9.4	2.2	
Gestational Diabetes			0.10			0.22			0.14
Missing	8.3	2.1		9.7	2.5		9.7	1.2	
No	9.5	2.6		9.5	2.9		9.9	2.7	
Yes	10.0	2.6		9.9	3.0		10.3	2.9	
Pre-eclampsia			0.067			0.023			0.19
Missing	9.0	2.2		9.6	2.5		9.8	1.6	
No	9.6	2.6		9.6	2.9		10.0	2.7	
Yes	9.1	2.2		8.8	2.4		9.5	2.6	
Delivery			0.0021			0.102			0.024
Pre-Term	8.8	2.2		8.9	2.9		9.3	2.8	
Full Term	9.6	2.6		9.5	2.9		10.0	2.7	
Gestational 25(OH)D			<0.0001			<0.0001			<0.0001
<20 ng/dL	9.1	2.4		8.9	2.5		9.4	2.4	
20.00–29.99 ng/dL	9.8	2.8		9.7	3.0		10.2	2.9	
≥30 ng/dL	10.1	2.4		10.5	3.2		10.4	2.6	

^1^ *p*-value for comparison among the subgroups (*i.e.*, race, insurance, *etc.*) for cognitive, expressive, or receptive scaled scores.

### 3.3. Modeling of Gestational 25(OH)D with Cognitive and Language Assessments at Age 2

In univariate analyses, gestational 25(OH)D was positively associated with cognitive scaled scores, receptive language, and expressive language (*p* < 0.001; Table 3). In multivariate analyses—when controlling for tobacco use during pregnancy, maternal IQ, education, race, child’s gestational age at birth and age at the 2-year exam—gestational 25(OH)D status was positively associated with receptive language scaled scores (*p* < 0.017) (Table 3), but not with cognitive or expressive language scaled scores. Applying race-stratified analyses, we obtained similar results for each race; however, statistical significance was not reached at the classical 0.05 type-I error rate due to the reduced sample size in the EA group.

**Table 3:** Relationships between 10 ng/dL increase for serum 25(OH)D during 2nd trimester with cognitive and language scaled scores ^1^.

Scaled Score *n* = 1020	Unadjusted Estimate	Unadjusted *p*	Adjusted Estimate	SE	Adjusted *R*^2^	Adjusted *p*
Cognitive	0.51	<0.001	0.07 ^2^	0.01	0.23	0.45
Receptive Language	0.71	<0.001	0.24 ^3^	0.10	0.21	0.017
Expressive Language	0.45	<0.001	0.12 ^4^	0.09	0.13	0.22

^1^ Bayley Scales of Infant and Toddler Development^®^, 3rd Edition (Bayley-III^®^) [34]; ^2^ Adjusted for tobacco use during pregnancy, total number of pregnancies, maternal IQ, gestational age at birth, and race; ^3^ Adjusted for tobacco use during pregnancy, maternal IQ, insurance, race, child age at CV2 clinic visit, and race; ^4^ Adjusted for tobacco use during pregnancy, maternal IQ, education, and race.

## 4. Discussion

To our knowledge, this is the largest prospective study to date examining the potential impact of gestational 25(OH)D status upon early childhood cognitive and language development in a racially diverse population. We found that higher gestational 25(OH)D status significantly associated with higher scaled scores for receptive language in offspring at 2 years of age. Similarly, positive trends were suggested between gestational 25(OH)D status and the scaled scores for cognition and expressive language, although observations did not reach statistical significance. Controlling for maternal IQ, race, or use of tobacco products during pregnancy reduced the effect size of 25(OH)D on scaled scores at age 2 years, underscoring the importance of capturing and evaluating multiple exposures when studying neurocognitive development.

Neurocognitive assessments in early childhood assess brain development, which begins with formation of the neural plate 12 days post-conception and continues throughout early adulthood. Brain structure and function can be affected by nutrient insufficiencies or environmental insults at critical time points. Animal studies provide biological plausibility for the hypothesis that *in utero* vitamin D exposure affects human brain development [39,40]. Expression of vitamin D receptors (VDR) in mammalian brain occurs as early as day 12 of gestation, then increases throughout pregnancy [40]. The presence of 1α-hydroxylase activity within numerous brain regions suggests that brain tissue can locally produce metabolically active vitamin D, further implicating a role for vitamin D in brain development. Murine dams maintained on vitamin D deficient diets before and during pregnancy display alterations in murine brain morphology that could contribute to deficits in memory, learning, and attention processing [39].

In humans, 25(OH)D levels during pregnancy have been positively associated with neurocognitive development in some [14,16] but not all studies [40,41,42]. Our observations support that language development is positively associated with gestational 25(OH)D levels as early as 2 years, while Whitehouse *et al.*, showed an effect as late as age 7 [14]. Other groups have not reported strong associations with gestational 25(OH)D [16,41,42,43], using a composite score to represent overall development. Combining cognitive and language development into one index could mask an association if it exists for a sub-scale. Our study and that of Whitehouse *et al.* [14] independently examined the associations with cognitive and language, and the available findings support a role for gestational 25(OH)D status in early language development.

In the current study, the small effect sizes for the association between gestational 25(OH)D status and receptive language scaled scores agree with reports by other investigators [40]. While stringent statistical significance was lacking for the cognitive score, the associations for all scaled scores trended consistently positive. In practical terms, an effect estimate of 0.07 for cognitive scaled score per 10 ng/dL increase in mid-gestational 25(OH)D translates to an increase of 0.35 IQ point for the 2-year-old child [44]. Similarly, an effect estimate of 0.24 for receptive and 0.12 for expressive language scaled score per 10 ng/dL increase in 25(OH)D translates into an increase of 1.0 and 0.6 IQ point respectively by age 2. The reduction in lifetime earnings has been estimated at $22,000 lost for each 1 IQ point decrease [44]. If the observed deficit in receptive language is maintained as the CANDLE children develop, the collective loss of total earnings would be estimated at $19 million. If the deficit in receptive language scaled scores increases as these children age, the total loss of earnings could be considerably more.

For optimal development, brain cells require a complement of macro- and micronutrients [2,45]. When specific nutrients are limiting, the brain has priority use at the expense of other organs [2,45]. Since the current inquiry focused on gestational 25(OH)D as a biomarker of nutrient exposure, it is possible that a peripheral marker is limited as an assessment of the availability of 25(OH)D for brain development. Animal data suggest the genetic expression of *Fox2*, a regulator of speech and language, was lower in those on the vitamin D deficiency diet at 14.5 days but expression was higher at 17.5 days, compared to those on a vitamin D sufficient diet [39]. These findings suggest that some compensatory mechanisms may exist to minimize effects of vitamin D deficiency.

Brain development from conception to age 5 varies across neurocognitive domains [46]. During sensitive stages of development, nutrient deficiency has adversely affected early child development [2,45] with persistent effects into adulthood [3]. The interaction of nutrient influences and socio-economic factors can be difficult to parse out in developmental studies [47,48]. We chose to use insurance as a measure of income because 9% of our sample did not report income, but did report insurance status. 99% of those not reporting income reported to be on Medicaid (TennCare) insurance. Income was collected in categories with the highest income category as ≥$75,000. Limitations due to how the income variable was obtained prevented the calculation of income as a continuous function of the federal poverty level. SES is a complex variable comprised of income, wealth, education, marital status, location of primary residence, and other factors. Based on the data available, we evaluated SES through a combination of variables (*i.e.*, insurance as a proxy for income, marital status, education, and maternal IQ). Our final multivariate models support that SES is an important component of cognitive and language development. With regard to cognitive and expressive language, SES accounted for the most of the variability in the model, resulting in little association with 25(OH)D.

Two other limitations exist for this study design. First, tobacco use and alcohol use during pregnancy information were collected as dichotomous variables (“yes/no”) by questionnaire, which could have affected the low rates. Using dichotomous variables merges low and moderate-heavy users. Effects of low levels of smoking and alcohol use by many women may mask effects of moderate to heavy use by a smaller group of users. In multivariate modeling of this CANDLE sample, alcohol was not found to be a significant covariate, after accounting for race, education, mother’s IQ, and mother’s tobacco use. Thus, the variability associated with alcohol use was taken up by these other variables and not important in the final model. To determine whether smoking and alcohol use during pregnancy affect subsequent cognitive outcomes, future studies could collect these data using continuous measures of exposure (e.g., how many cigarettes did the mother use per day and how much alcohol did she consume?).

Second, the Bayley Scales detect general milestones and are less sensitive to subtle effects than narrow band assessments, such as continuous measures of recognition memory, symbolic play, acuity, or information processing speed. Despite these limitations, impressive effects on receptive language were observed. These findings suggest that this may be the tip of the iceberg and that other effects might be detected if more sensitive narrow band tests are used.

In our preliminary evaluation, we were unable to find any association with diet-based nutrient influences on neurocognitive development. Our bivariate data suggest that 25(OH)D sufficiency, maternal education level, ability to buy health insurance, use of tobacco products, premature birth, and marital status all affected the cognitive and language development at age 2. When multivariate analyses include the possible confounders, the effect size of gestational 25(OH)D on language was reduced in our study, in agreement with other reports [16]. The positive associations between *in utero* 25(OH)D and language development remained, underscoring the consistency of findings across multiple cohorts.

Unlike other studies, our cohort was racially diverse, thereby allowing us to examine the effects of 25(OH)D in a population more representative of the USA. Differences in cognitive and language scaled scores were present between our AA and EA children. IQ scores favoring EA over AA have been documented [49,50,51,52,53]. Our 1-point difference in scaled score between AA and EA is equivalent to that reported by other groups [51,54]. Although controversial, the discussion often focuses on the “nurture *versus* nature” that may lead to this difference [49,50,51,52,55,56]. We recently reported [57] that DNA methylation is jointly modulated by ancestry and gestational vitamin D levels, observations which support multidimensional factors. Despite these arguments, our data suggest the association between gestational 25(OH)D and receptive language are not different between AA and EA, when adjusted for other SES and maternal characteristics.

Strengths of our study include its contemporary design, large size, high retention rate (75% of the total CANDLE cohort attended the 2-year exam), permanent residence limited to one county (to reduce potentially confounding effects of latitude on sunlight exposure), high number and high proportion of African-American participants, and uniformity of data collection techniques throughout the study. Due to phlebotomy limits in infants and the burden of other CANDLE procedures, this CANDLE sub-study lacks repeated measures of 25(OH)D status for participants and lacks individual assessments of parathyroid hormone status or vitamin D receptor (*VDR*) genotype. The design also could not control for potential confounding by differences in sunscreen use, sun exposure (e.g., air pollution or outdoor lifestyles), culture, skin cover by clothing, or post-natal vitamin D intake via diet. Lower gestational 25(OH)D levels perhaps reflected dietary intake during pregnancy or less intense sun exposure in Memphis (latitude range 35° N–35°24′ N) compared to more equatorial latitudes.

## 5. Conclusions

Higher *in utero* 25(OH)D exposures during the second trimester were positively associated with receptive language skills in both EA and AA toddlers at age 2 years. Once we adjusted for other environmental factors, this contribution of gestational 25(OH)D status remained in the final models. Whether these small differences and disparities persist and/or increase throughout childhood remains to be determined.

## Abbreviations

Conditions Affecting Neurocognitive Development and Learning in Early Childhood Study, CANDLE; Bayley Scales of Infant and Toddler Development, Third Edition, BSID III; Wechsler Abbreviated Scale of Intelligence, WASI; 25-hydroxy-vitamin D, 25(OH)D; body mass index, BMI; vitamin D binding protein, VDBP.
